# Taraxacum Mongolicum Polysaccharides Reverses Mice Obesity via Activation of AKT/mTOR Pathway

**DOI:** 10.3390/nu16193330

**Published:** 2024-09-30

**Authors:** Xiaoyu Yue, Shilong Yu, Yue Luan, Jianpeng Wang, Junxing Zhao, Mu Zhang, Qin Wang

**Affiliations:** 1 State Key Laboratory of Animal Biotech Breeding, College of Animal Science and Technology, China Agricultural University, Beijing 100193, China; b20233040387@cau.edu.cn (X.Y.); slyu@cau.edu.cn (S.Y.); luanyue@cau.edu.cn (Y.L.); 15074813960@163.com (J.W.); 2 College of Animal Science, Shanxi Agricultural University, Jinzhong 030801, China; junxzh@sxau.edu.cn; 3 College of Economics and Management, Shenyang Agricultural University, Shenyang 110065, China; zm8842@sina.com

**Keywords:** Taraxacum mongolicum polysaccharides, obesity, adipose tissue browning, AKT, mTOR

## Abstract

Background/Objectives: The global prevalence of obesity and its associated health complications represent significant public health concerns. Plant polysaccharides have been demonstrated to possess a range of beneficial pharmacological effects. This experiment was designed to elucidate the mechanisms of dietary Taraxacum mongolicum polysaccharides involved in the regulation of obesity and fat browning. Methods: Male C57BL/6J mice were randomly divided into three groups: a control group, a high-fat diet (HFD) group, and an HFD group supplemented with 0.3% TMPs. The mice were fed their respective diets for 10 weeks, after which their body weight, food consumption, and serum lipid levels were measured. Histological analysis was performed to assess lipid deposition in adipose tissue and liver. Western blot was used to assess the expression of proteins involved in the AKT/mTOR pathway. Results: The results show that compared with the HFD group, the TMP supplementation group’s body-weight gain (12.17 ± 1.77) significantly decreased. TMPs also reduced serum levels of total cholesterol, triglycerides, and low-density lipoprotein cholesterol. Histological analysis showed that TMPs reduced lipid deposition in both adipose tissue and the liver. Conclusions: In addition, TMPs increased the expression of phosphorylated AKT and the mechanistic target of rapamycin (mTOR), indicating that TMPs exert their beneficial effects on lipid metabolism via the AKT/mTOR pathway.

## 1. Introduction

Lipid deposition, also referred to as fat accumulation, is a significant global health concern due to its association with various chronic diseases, including obesity, type 2 diabetes, and cardiovascular diseases [1]. The detrimental effects of an HFD on human health have been extensively documented in the scientific literature. A substantial body of evidence from epidemiological and experimental studies demonstrates that such diets can contribute to the development of a range of chronic diseases, including obesity, type 2 diabetes, cardiovascular disease, and non-alcoholic fatty liver disease [2]. Various pharmacological strategies, such as sibutramine, fluoxetine, sertraline, orlistat and topiramate, have been devised for addressing obesity [3]. Nonetheless, their administration demands careful consideration owing to potential adverse effects and the potential for substance misuse [4].

Recent studies have highlighted the potential of polysaccharides from various sources, including Taraxacum mongolicum, in combating obesity [5,6,7]. The specific anti-obesity effects of TMPs are likely due to their ability to reduce body weight gain, improve insulin resistance, and regulate glycolipid metabolism. In an experiment conducted by Zhang et al. [8], dandelion extract was administered to mice. The extract exhibited anti-obesity potential, demonstrating a dose-dependent inhibition of pancreatic lipase activity and an increase in plasma triglyceride levels. In a further study, the anti-obesity effects of dandelion ethanol extract were examined. The administration of dandelion ethanolic extract orally at doses of 150 and 300 mg/kg was found to significantly reduce body weight, lipid parameters, organ weight and fat pad mass [9]. It is regrettable that it is not feasible to ascertain which compounds are accountable for this effect, given that the concentrations of phytoactive compounds in the extracts have not been quantified.

The AKT/mTOR pathway is a signaling pathway that plays a crucial role in regulating cell growth, proliferation, and metabolism. It has been implicated in the regulation of adipogenesis and energy homeostasis. Pena-León [10] and Fernández-Veledo [11] both highlight the involvement of mTOR in energy balance and adipocyte differentiation. Shearin [12] further emphasizes the significance of AKT in adipocyte function and systemic metabolism, with Kwan [13] specifically noting its role in glucose homeostasis. These findings underscore the importance of the AKT/mTOR pathway in maintaining energy balance and regulating adipogenesis.

Brown adipose tissue (BAT) is a metabolically active fat tissue that plays a crucial role in energy expenditure and glucose homeostasis [14]. The transformation of white adipose tissue to BAT, known as browning, has emerged as a promising strategy for treating obesity and related metabolic disorders [15]. BAT activation can increase energy expenditure and promote a negative energy balance, potentially alleviating metabolic complications like dyslipidemia and insulin resistance [16]. Furthermore, the induction of browning in white adipose tissue has been suggested as a novel therapeutic strategy for obesity and type 2 diabetes mellitus [17].

Recent studies have demonstrated that the mTOR signaling pathway plays a pivotal role in the regulation of adipose tissue functions, including adipogenesis, lipid metabolism, thermogenesis, and the synthesis and secretion of adipokines. The enhancement of adipose mTOR signaling by adipose-specific pathways resulted in a reduction in core body temperature and cold tolerance in mice, as well as an attenuation of cold-induced thermogenic gene expression in both BAT and inguinal WAT. In contrast, WAT, but not BAT, of fat-specific raptor-knockout mice exhibited elevated expression of Ucp1, type 2 deiodinase (Dio2), and Cidea, indicating that the mTOR signaling pathway exerts a negative regulatory effect on the development of beige fat. Given the pivotal role of the mTOR signaling pathway in regulating energy homeostasis, we postulated that TMPs may enhance lipid deposition in HFD-induced obese mice via the AKT-mTOR pathway.

## 2. Materials and Methods

### 2.1. Ethics Statement

Thirty healthy male C57BL/6J mice (6 weeks old, weighing 23.1 ± 1.4 g) were purchased from SPF Biotechnology Co., Ltd. (Beijing, China). The mice were randomly divided into three groups: a normal diet group (control group; SPF-F02, SPF Biotechnology Co., Ltd., Beijing, China), a high-fat diet group (HFD group; 60% energy from fat, D12492; SPF Biotechnology Co., Ltd., Beijing, China), and an HFD group supplemented with 0.3% TMPs (HFDTMP group; SPF Biotechnology Co., Ltd., Beijing, China) with 10 mice in each group. Recently published data on the dandelion polysaccharides ameliorating high-fat-diet-induced atherosclerosis in mice through antioxidant and anti-inflammatory capabilities were used as a basis for sample size calculation [18]. TMPs (Ksm100) were purchased from Shaanxi Ciyuan Biotechnology Co., Ltd. (Xi’an, China). The animals were housed individually in cages and kept under a 12:12 h light/dark cycle. Animals were randomized to groups after acclimation using a computer-based random sequence generator. Animals were distributed in 6 cages (5 mice per cage, 2 cages per treatment group). For each animal, the researcher in charge of keeping the animal was the only person who knew the experimental group assignment. No other participants knew the group assignment. The animal experiments presented in this study were conducted in accordance with the guidelines of the animal welfare committee of the State Key Laboratory for Agro-Biotechnology of the China Agricultural University (approval number, AW72203202-1-1; date, 27 February 2023).

### 2.2. Glucose Tolerance Tests (GTT) and Insulin Tolerance Tests (ITT)

Prior to the commencement of the experiment, the mice were fasted for a period of 12 h. Following this, they were intraperitoneally injected with D-glucose (2 g/kg body weight). Blood samples were collected at 30, 60, 90, and 120 min intervals after the injection of the glucose solution. The ITT is comparable to the GTT in that mice are fasted overnight before testing. Subsequently, the mice were intraperitoneally injected with insulin (1 U/kg body weight). Blood glucose concentrations were tested at 30, 60, 90, and 120 min post-injection using a glucometer (Sinocare Inc. Co., Ltd., Changsha, China).

### 2.3. Rectal Temperature and Infrared Image

In the 10th week of the experiment, a test was conducted to induce thermogenesis in response to cold. The mice were transferred to fresh cages and deprived of both food and water. Subsequently, the mice were exposed to cold stimulation at a temperature of 4 °C for 12 h. Rectal core temperature was measured using a digital thermometer (Physitemp Instruments, Clifton, NJ, USA), while the temperature of BAT was recorded with a FLIR E6xt camera (FLIR Systems OÜ, Tallinn, Estonia).

### 2.4. Serum Inflammatory Marker Measurement

After collection, blood samples from each mouse were centrifuged at 3000 rpm for 10 min. The resulting plasma was promptly frozen at −80 °C until analysis. Mouse serum inflammatory marker levels were quantified using an ELISA Kit (Invitrogen, Carlsbad, CA, USA) following the manufacturer’s guidelines.

### 2.5. Hematoxylin and Eosin Staining (H&E Staining)

Small pieces of adipose and liver were randomly selected and fixed in a 10% neutral formalin solution for 24 h. The samples were subsequently washed with water and dehydrated using an ethanol gradient for 30 min: 50%, 70%, 85%, 95%, and 100% ethanol. Following dehydration, the samples were treated with xylene to remove the dehydrating agent. The tissue sections, with an average thickness of about 5μm, were embedded in paraffin for HE staining and pathological observation. To begin the dewaxing process, we used xylene twice for 10 min each time. We proceeded to wash the sample with gradient ethanol of 100%, 95%, 85%, 70%, and 50%, followed by a rinse with deionized water for 5 min. The tissue sample was stained with hematoxylin for 10 min, followed by a 5 min wash with water. Next, it was differentiated with 1% hydrochloric acid ethanol for 30 s and washed with water for another 30 s. The sample was then stained with 0.5% eosin for 2 min and rinsed with deionized water for 1 min. To dehydrate the sample, a gradient ethanol series was used, starting with 70% ethanol for 1 min and followed by 85% ethanol for 1 min, 95% ethanol for 3 min, and 100% ethanol for 3 min, twice. Finally, the sample was immersed in xylene for 10 min and sealed with neutral gum. Subsequently, 10 randomly selected fields of view were captured for each sample using a microscope (DMi8, Leica, Wetzlar, Germany).

### 2.6. RNA Exaction, cDNA Synthesis, and Quantitative Real-Time PCR (qRT-PCR)

Total RNA was isolated from mouse tissue using TRIzol (Sigma, Saint Louis, MO, USA) following the instructions. RNA quality was assessed using a NanoDrop instrument (ND-2000, Thermo Scientific, Rockfork, IL, USA). The isolated mRNA was reverse transcribed into cDNA using a reverse transcription kit (RR036B, TaKaRa Biotechnology Co., Ltd., Dalian, China). The real-time fluorescence quantitative PCR reaction system (20 μL) consisted of 2 × SYBR qPCR Mastermix 10 μL, Primer forward 0.4 μL, Primer reversed (10 μm) 0.4 μL, Template DNA 1 μL, and DEPC water 8.2 μL. The above system was reacted in a CFX Connect Real-time PCR Detection System (Bio-Rad, Hercules, CA, USA), and the PCR reaction program was as follows: 95 °C for 30 s, 95 °C for 10 s, 60 °C for 20 s, and 72 °C for 60 s, 40 cycles. The primer sequences of related genes in this experiment are shown in Table 1. 

### 2.7. Western Blotting

In order to extract proteins from the organization, 20 mg of each sample was taken and added to 100 μL of RIPA lysis buffer containing a final concentration of 1 mmol·L-1 PMSF (Solarbio, Beijing, China). The mixture was then placed on ice and left to lyse for 30 min. After completion of the lysis process, the sample was centrifuged at 12,000× *g* for 5 min at 4 °C. The resulting supernatant was transferred to a microcentrifuge tube and stored at −20 °C for future use. Protein quantification was performed using the BCA protein concentration assay kit. A certain amount of protein sample was mixed with the loading buffer and boiled at 95–100 °C for 5 min. The protein was then separated using 10% polyacrylamide gel electrophoresis. The gel was concentrated at 80 V for 30 min, followed by separation at 120 V for 60 min. After electrophoresis, the separated proteins were transferred onto a PVDF membrane using a transfer apparatus with a current of 320 mA for 60 min. 

The membrane was then blocked with blocking solution (20 mL TBST, 1 g skim milk powder) for 1 h. The appropriate primary antibody was added and incubated overnight at 4 °C. The membrane was removed from the primary antibody solution and washed with TBST four times for 6 min each time. The appropriate secondary antibody was then added and incubated at room temperature for 1 h. After incubation with the secondary antibody, the membrane was washed with TBST four times for 6 min each time. Finally, the target protein was detected using the Odyssey Infrared Imaging System (LI-COR Biosciences, Lincoln, NE, USA). 

The following primary antibodies were used: CCAAT/enhancer-binding protein β (C/EBP β; 1:1000, bs1396R), PR domain-containing 16 (PRDM16), UCP1 (1:1000, bs1925R), and β-actin (1:20,000, bs-0061R) (all from Biosynthesis Biotechnology Co., Ltd., Beijing, China), and anti-PGC1α (1:1000, ab54481) were obtained from Abcam (Cambridge, MA, USA). Anti-Akt (1:1000, 9272), anti-phospho-AKT (1:1000, 9271), anti-mTOR (1:1000, 2972), and anti-phospho-mTOR (1:1000, 2971) were obtained from Cell Signalling Technology (Danvers, MA, USA). The secondary antibody, goat anti-rabbit IgG-HRP (1:5000, BA1054), was obtained from Boster (Wuhan, China). The relative expression levels of proteins were reflected by the gray ratio and normalized to the β-actin content in each group.

### 2.8. Statistical Analysis

Statistical analysis of the obtained data was performed using the unpaired Student’s *t*-test, one-way analysis of variance (ANOVA), and Holm–Sidak multiple comparison test with correction. Tukey’s honestly significant difference test (Tukey’s HSD) was employed for the purpose of conducting pairwise mean comparisons. We generated charts using GraphPad Prism 7.0. Data are presented as mean ± standard error of the mean (SEM), where *p* < 0.05 is considered significant and *p* < 0.01 is considered extremely significant.

## 3. Result

### 3.1. TMPs Alleviate HFD-Induced Obesity in Mice

**Figure 1:**
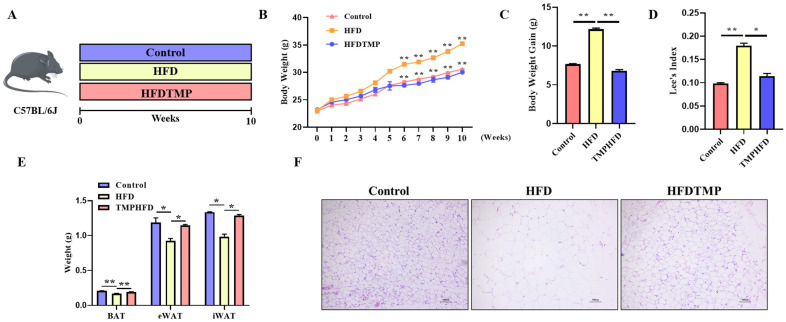
Effects of TMPs on obesity in mice. (**A**) Schematic diagram of mouse feeding. (**B**) Weekly body weight. (**C**) Body weight gain. (**D**) Lee’s index. (**E**) Weight of BAT, eWAT, and iWAT. (**F**) H&E staining of WAT sections. Scale bars indicate 100 μm. (Mean ± SEM; *n* = 10 in each group; * *p* < 0.05, ** *p* < 0.01).

To investigate the impact of TMPs on obesity, mice were fed an HFD, an HFD supplemented with 0.3% TMPs (HFDTMP), or a control diet for 10 weeks (Figure 1A). The mice on the HFD had a higher body weight than those in the control group. However, this disparity was mitigated with TMP supplementation (Figure 1B,C, *p* < 0.01). Since Lee’s index is one of the effective indices for evaluating the degree of animal obesity, Lee’s index of mice was calculated. Lee’s index was significantly reduced in the HFDTMP group compared to the HFD group (Figure 1D, *p* < 0.05). The HFD-fed mice showed increased accumulation of intra-abdominal fat, which was notably attenuated by TMP intake. Furthermore, although the HFD led to elevated weights of BAT, eWAT, and iWAT, those in the HFDTMP groups approached the average weight of control mice (Figure 1E, *p* < 0.05). There were no significant differences in food intake between the three groups (Figure S1). Additionally, histological examination using H&E staining revealed that mice supplemented with TMPs exhibited adipocyte sizes in WAT (Figure 1F).

### 3.2. Administration of TMPs Maintains Glucose Homeostasis

**Figure 2:**
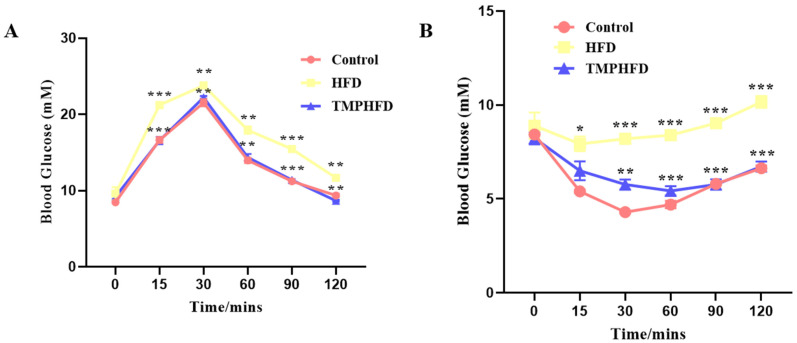
Effects of TMP on GTT and ITT in mice. (**A**) Blood glucose levels following GTT test. (**B**) Blood glucose levels following ITT test. (Mean ± SEM; *n* = 10 in each group; * *p* < 0.05, ** *p* < 0.01, *** *p* < 0.001).

The GTT results indicate that mice in the HFDTMP group exhibited better glucose tolerance compared to those in the HFD group (Figure 2A). Additionally, the ITT results showed an increase in insulin sensitivity among mice in the HFDTMP group (Figure 2B).

### 3.3. TMPs Counteract HFD-Induced Hepatic Steatosis

**Figure 3:**
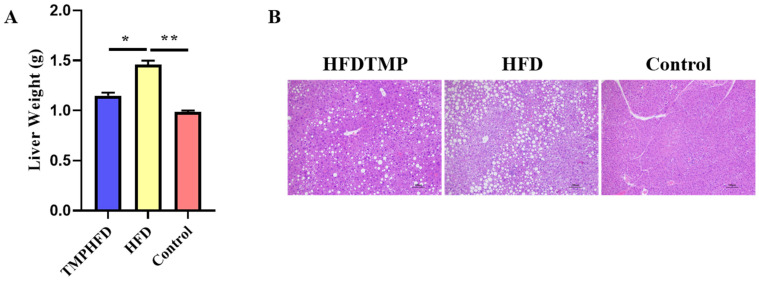
Effects of TMPs on hepatic steatosis in mice. (**A**) Weight of liver. (**B**) H&E staining of liver sections. Scale bars indicate 100 μm. (Mean ± SEM; *n* = 10 in each group; * *p* < 0.05, ** *p* < 0.01).

The consumption of an HFD resulted in an increase in liver weight, which was effectively reversed by the supplementation of TMPs (Figure 3A). Histological examination using H&E staining indicated that the consumption of an HFD caused abnormal triglyceride accumulation in the liver. However, mice supplemented with HFDTMP exhibited minimal lipid deposition (Figure 3B). As expected, feeding mice an HFD for 10 weeks resulted in hyperlipidemia, which was characterized by decreased levels of high-density lipoprotein cholesterol (HDL-C) and elevated levels of low-density lipoprotein cholesterol (LDL-C), triglycerides [19], and total cholesterol (TC) in both serum and liver (Table 2 and Table 3, respectively). It is worth noting that compared to the HFD group, the HFDTMP group showed a significant improvement in hyperlipidemia (Table 2).

### 3.4. TMPs Attenuate HFD-Induced Inflammation in Mice

**Figure 4:**
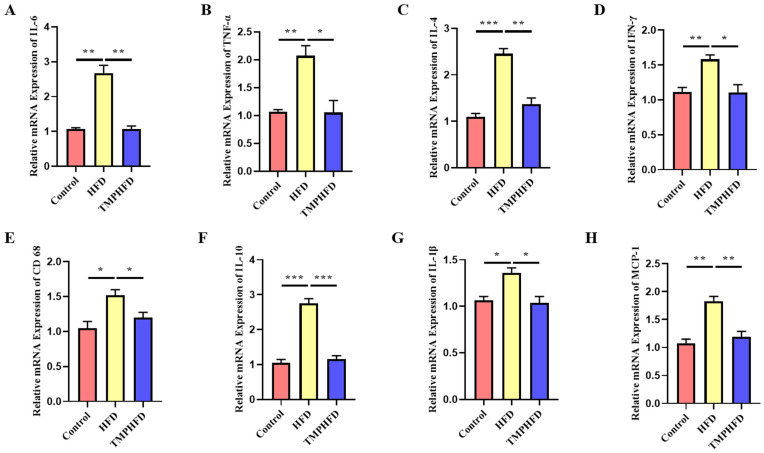
Effect of TMPs on inflammation in mice. (**A**–**H**) mRNA contents of IL-6, TNF-α, IL-4, IFN-γ, CD 68, IL-10, IL-1β, and MCP-1 in iWAT, respectively (mean ± SEM; *n* = 8 in each group; * *p* < 0.05, ** *p* < 0.01, *** *p* < 0.001).

To investigate the impact of TMPs on inflammation in mice, we employed ELISA, RT-qPCR, and Western blotting to measure levels of inflammatory markers. The ELISA results showed increased levels of interleukin 6 (IL-6), interleukin 10 (IL-10), and tumor necrosis factor α (TNF-α) in the serum of the HFD group mice, while TMP supplementation significantly reduced HFD-induced inflammation (Table 4). Additionally, TMP administration resulted in a significant decrease in mRNA expression of IL-6, TNF-α, interleukin 4 (IL-4), IL-1β, interferon-γ (IFN-γ), CD 68, interleukin 1β (IL-1β), and monocyte chemoattractant protein-1 (MCP-1). Furthermore, the protein expression levels of these markers were significantly reduced compared to the control group (Figure 4A–H). Overall, our findings indicate that TMPs may primarily alleviate inflammation by suppressing the expression of these genes.

### 3.5. TMP Intake Restores BAT Function in HFD-Induced Obesity

**Figure 5:**
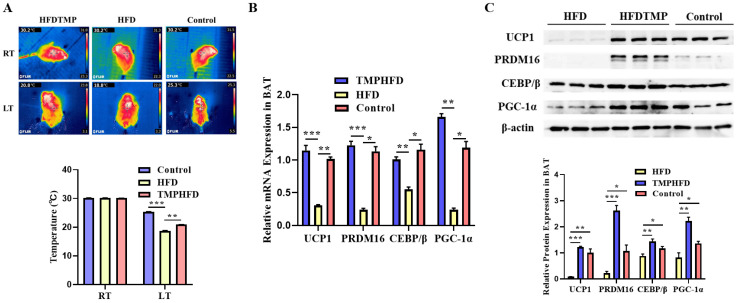
Effect of TMPs on BAT function in mice. (**A**) Thermal images. (**B**) Relative mRNA expression of UCP1, PRDM16, C/EBP β, and PGC-1α in the different groups. (**C**) Protein expression of UCP1, PRDM16, C/EBP β, and PGC-1α in the different groups. (mean ± SEM; *n* = 8 in each group; * *p* < 0.05, ** *p* < 0.01, *** *p* < 0.001).

Figure 5A shows that there were no noticeable differences in body surface temperature between the groups before cold exposure. However, after cold stimulation, the mice that were fed the HFD had significantly lower surface temperatures compared to the chow group (*p* < 0.01). The decline was effectively mitigated by TMP supplementation (*p* < 0.05). In comparison to control mice, the BAT of mice fed an HFD exhibited a phenotype that was similar to white fat. However, dietary TMPs significantly reduced lipid accumulation in BAT (Figure 5B). Additionally, TMP intake restored the decreased mRNA and protein levels of PRDM16, UCP1, PGC-1α, and CEBP/β in the BAT of HFD-fed mice (Figure 5C, *p* < 0.01).

### 3.6. Dietary TMP Supplementation Potentiated Beige Adipogenesis

**Figure 6:**
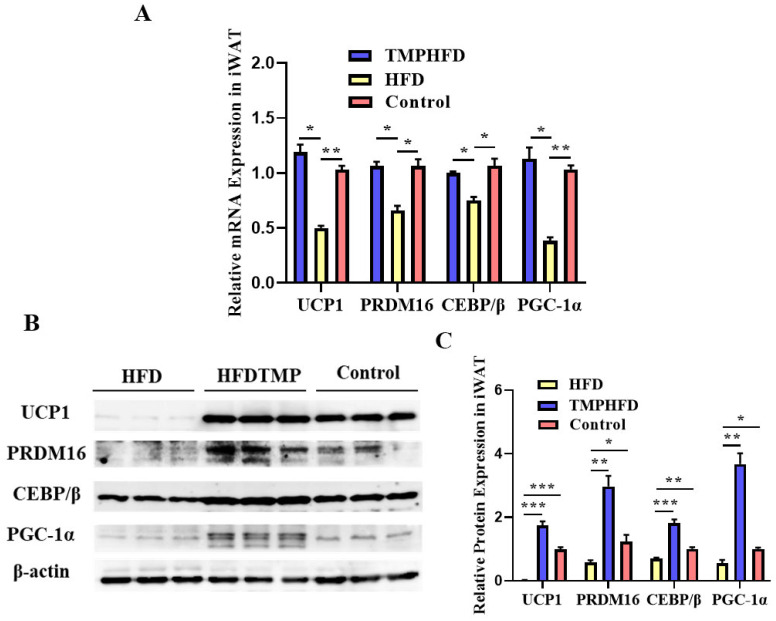
Effect of TMPs on beige adipogenesis in mice. (**A**) Thermal images. (**B**) Relative mRNA expression of UCP1, PRDM16, C/EBP β, and PGC-1α in the different groups. (**C**) Protein expression of UCP1, PRDM16, C/EBP β, and PGC-1α in the different groups. (mean ± SEM; *n* = 8 in each group; * *p* < 0.05, ** *p* < 0.01, *** *p* < 0.001).

To evaluate the potential of TMPs in promoting beige adipogenesis in iWAT, we conducted histological analysis. Our findings revealed a greater accumulation of multilocular beige adipocytes in the iWAT of the HFDTMP group (Figure 6A, *p* < 0.05). Additionally, the mRNA expression of the thermogenic gene was notably elevated in the iWAT of mice supplemented with TMPs (Figure 6B, *p* < 0.05), indicating enhanced formation of beige adipocytes. This observation was further supported by alterations in key protein contents, including UCP1, PRDM16, PGC-1α, and CEBP/β, which confirmed the increased formation of beige adipocytes in the iWAT of the HFDTMP group.

### 3.7. TMPs Induce Browning through AKT/mTOR-Thermogenesis Pathway in Both BAT and iWAT

**Figure 7:**
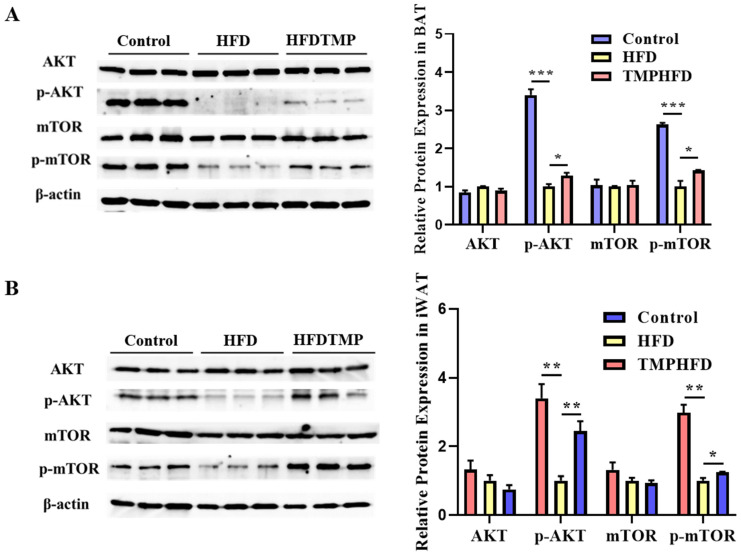
Effect of TMPs on AKT/mTOR-thermogenesis pathway in mice. (**A**,**B**) Protein expression of AKT, mTOR, *p*-AKT, and *p*-mTOR in the different groups in both BAT and iWAT (mean ± SEM; *n* = 8 in each group; * *p* < 0.05, ** *p* < 0.01, *** *p* < 0.001).

The results showed reduced levels of the *p*-AKT and *p*-mTOR protein in both BAT and iWAT of HFD-fed mice (Figure 7A,B, *p* < 0.05). TMP treatment notably alleviated this inhibition, and the relative protein levels of *p*-AKT and *p*-mTOR were restored in both BAT and iWAT in the HFDTMP group (Figure 7A,B, *p* < 0.05).

## 4. Discussion

Obesity and its associated health complications are a global concern, leading to extensive research into effective weight management strategies [20]. One promising avenue is exploring the potential of natural compounds to regulate body weight and metabolic processes [21]. Although previous research has indicated that Taraxacum mongolicum extract possesses anti-obesity properties in animal models, the key components responsible for its efficacy and its mechanism of action remain unclear [22]. In this context, our study utilizes a mouse model, specifically male C57BL/6J mice categorized into three groups: a control group, an HFD group, and an HFDTMP group. Over a period of 10 weeks, various parameters, such as body weight, food consumption, serum lipid levels, and histological changes in adipose tissue and liver, are measured. The focus is on understanding how TMP supplementation affects these factors and whether it can be linked to specific molecular pathways. 

The study investigated the potential effects of TMPs on obesity in mice subjected to an HFD. The significant reduction in body weight gain and improved insulin sensitivity and glucose tolerance observed in mice on an HFD supplemented with TMPs suggest a potential role for TMPs in weight management and glucose homeostasis maintenance. Obesity frequently results in the development of type 2 diabetes as a consequence of systemic insulin resistance. The results of both the GTT and ITT demonstrated that the HFD induced insulin resistance in the mice. However, the addition of TMPs significantly increased blood glucose clearance, suggesting that TMPs may act as an insulin sensitizer, possibly due to their ability to inhibit α-glucosidase [23]. Alpha-glucosidase is an enzyme responsible for breaking down complex carbohydrates (disaccharides, oligosaccharides, and polysaccharides) into simple sugars (including glucose). By inhibiting the breakdown of alpha bonds in carbohydrates, alpha-glucosidase inhibitors reduce glucose absorption from the bloodstream from the gastrointestinal tract, thereby lowering postprandial blood sugar. In addition, researchers could observe insulin secretion from INS-1 cells in dandelion extract at a concentration of 40 µg/mL [24]. Systemic insulin resistance accelerates the progression of non-alcoholic fatty liver disease, worsening insulin action and creating a harmful cycle. In this study, mice fed sand TMPs showed not only improved insulin sensitivity but also reduced HFD-induced hepatic steatosis. Fatty acid beta-oxidation in mitochondria is a process that breaks down fatty acids. Previous studies have found that bioactive compounds such as polysaccharides can enhance β-oxidation of fatty acids [25], suggesting that TMPs may contribute to the recovery of hepatic steatosis.

Moreover, TMPs exhibit a favorable impact on serum lipid levels, effectively reducing total cholesterol, triglycerides, and low-density lipoprotein cholesterol. Histological analysis further reveals a reduction in lipid deposition in adipose tissue and liver. Our findings are in line with prior research indicating that Taraxacum mongolicum alleviates the aberrant energy metabolism and lipid metabolism triggered by H_2_O_2_ [26].

Improving BAT function plays a significant role in weight management and obesity [27]. BAT is a metabolically active tissue that is specialized in dissipating energy as heat, thereby contributing to energy expenditure and thermogenesis [28]. When BAT function is enhanced, it can lead to increased energy expenditure, which may help in burning excess calories and reducing body weight [29]. One way to improve BAT function is through interventions such as dietary supplements or lifestyle modifications [30]. For example, certain compounds like thermogenic agents or nutrients like capsaicin and catechins have been shown to activate BAT and enhance its thermogenic activity [31]. The results of this study indicate the potential benefits of TMPs in improving BAT function. TMP supplementation alleviated the decrease in body surface temperature induced by the HFD, possibly indicating a role for TMPs in maintaining normal energy metabolism. TMPs reduced lipid accumulation in BAT, which may help maintain the brown adipogenic properties of BAT, thereby promoting energy expenditure. TMP intake restored the expression levels of key genes and proteins in BAT that were decreased by the HFD, further supporting the role of TMPs in maintaining BAT function. 

In our study, we explored the potential of dietary TMP supplementation in promoting beige adipogenesis in iWAT. Histological analysis revealed a greater accumulation of multilocular beige adipocytes in the iWAT of mice supplemented with TMPs in comparison to the control group. This suggests that TMPs have the ability to induce beige adipocyte formation within iWAT. Furthermore, mRNA expression of thermogenic genes was notably elevated in the iWAT of mice receiving TMP supplementation, indicating enhanced beige adipocyte formation. These findings were corroborated by alterations in key protein contents, including UCP1, PRDM16, PGC-1α, and CEBP/β, which are associated with beige adipocyte differentiation and function. The increased expression of these proteins further supports the notion that TMP supplementation potentiates beige adipogenesis in iWAT. Our results suggest that TMPs hold promise as a dietary supplement for promoting the formation of metabolically active beige adipocytes, which could have implications for combating obesity and improving metabolic health. 

In addition to promoting brown fat transformation and beige adipogenesis, TMPs have also been found to improve lipid metabolism and reduce inflammation in animal models of obesity. These findings suggest that TMPs may have potential as a therapeutic agent for the treatment of obesity and related metabolic disorders.

As a nutrient sensor and key regulator of energy metabolism, AKT/mTORx signaling plays an important role in the development and function of brown adipocytes [32]. To gain insights into the molecular mechanisms underlying these effects, the study employs Western blot analysis to assess the expression of proteins associated with the AKT/mTOR pathway. The observed increase in phosphorylated AKT and mTOR suggests that TMP treatment effectively counteracts the suppression of AKT and mTOR signaling pathways induced by HFD feeding. The restoration of these pathways may play a crucial role in mediating the beneficial effects of TMPs on metabolic homeostasis and adipose tissue function in the context of obesity.

A limitation of this study is that the feeding experiment was conducted for only 10 weeks, which may not have been sufficient to demonstrate the full extent of the observed improvement. Furthermore, only one concentration of TMPs was selected for the in vivo experiment. It is hoped that these limitations will be addressed in future studies.

In conclusion, the results of this experiment indicate the potential of TMPs as an obesity-modulating dietary intervention and provide a basis for further research into the specific molecular pathways involved. Nevertheless, further research, with a particular emphasis on clinical trials, is required to reinforce the association between TMPs and obesity and to ascertain the optimal dosage required to modulate these metabolic responses in humans.

## 5. Conclusions

TMPs have been shown to be effective in reversing obesity induced by an HFD and promoting brown fat transformation by activating the AKT/mTOR pathway. Further studies are needed to elucidate the molecular mechanisms underlying these effects and to determine the optimal dosage and duration of treatment. Nonetheless, these findings offer fresh perspectives on the possible application of TMPs as a nutritional intervention strategy for obesity.

## Figures and Tables

**Table 1:** Primer sequences for qRT-PCR.

Gene	Forward	Reverse
PRDM16	GCCTGTTTCTCTTCTGTCCCC	GCCAACAGGACGGTGTTATTT
UCP1	TTGCTTCTCTCAGGATCGGC	GTGGGTTGCCCAATGAACAC
C/EBP β	GCTGAGCGACGAGTACAAGATGC	CTTGTGCTGCGTCTCCAGGTTG
PGC-1α	TGTCGGATGCTTGCTTGAGT	TACGGTTGTAACGCAGGACCT
β-actin	AGGCCAACCGCGAGAAGATGACC	GAAGTCCAGGGCGACGTAGCAC

**Table 2:** Levels of HDL-C, LDL-C, TG, and TC in serum. (Mean ± SEM; *n* = 10 in each group; * *p* < 0.05, ** *p* < 0.01, *** *p* < 0.001).

Group	TC (mM)	TG (mM)	HDL (mM)	LDL (mM)
Control	8.91 ± 1.29	1.14 ± 0.17	0.66 ± 0.04	2.59 ± 0.11
HFD	13.82 ± 2.21 *	2.81 ± 0.12 ***	0.19 ± 0.03 ***	7.60 ± 0.28 ***
HFDTMP	8.01 ± 1.77 *	1.57 ± 0.31 **	0.35 ± 0.03 **	2.50 ± 0.21 ***

**Table 3:** Levels of HDL-C, LDL-C, TG, and TC in liver. (Mean ± SEM; *n* = 10 in each group; *** *p* < 0.001).

Group	TC (mM/g)	TG (mM/g)	HDL (mM/g)	LDL (mM/g)
Control	1.45 ± 0.04	0.43 ± 0.02	2.99 ± 0.14	0.95 ± 0.10
HFD	3.69 ± 0.12 ***	1.36 ± 0.04 ***	0.92 ± 0.08 ***	1.60 ± 0.07 ***
HFDTMP	1.66 ± 0.08 ***	0.41 ± 0.02 ***	2.85 ± 0.13 ***	1.03 ± 0.03 ***

**Table 4:** Serum levels of IL-6, IL-10, TNF-α, and COX-2. (Mean ± SEM; *n* = 10 in each group; * *p* < 0.05, ** *p* < 0.01, *** *p* < 0.001).

Group	IL-6 (pg/mL)	IL-10 (pg/mL)	TNF-α (pg/mL)	COX-2 (pg/mL)
Control	1.91 ± 0.29	0.41 ± 0.07	1.26 ± 0.14	0.95 ± 0.11
HFD	3.82 ± 0.21 ***	2.81 ± 0.11 ***	0.92 ± 0.08 *	2.6 ± 0.28 ***
HFDTMP	2.01 ± 0.17 ***	0.75 ± 0.09 ***	1.15 ± 0.13 *	1.5 ± 0.21 **

## Data Availability

The data presented in this study are available upon request from the corresponding author due to (specify the reason for the restriction).

## Supplementary Materials

The following supporting information can be downloaded at: https://www.mdpi.com/article/10.3390/nu16193330/s1, Figure S1.

## References

[1] Van Gaal L.F., Mertens I.L., De Block C.E. (2006). Mechanisms linking obesity with cardiovascular disease. Nature.

[2] Rohr M.W., Narasimhulu C.A., Rudeski-Rohr T.A., Parthasarathy S. (2020). Negative effects of a high-fat diet on intestinal permeability: A review. Adv. Nutr..

[3] Valentino M.A., Lin J.E., Waldman S.A. (2010). Central and peripheral molecular targets for antiobesity pharmacotherapy. Clin. Pharmacol. Ther..

[4] Edwards I.R., Aronson J.K. (2000). Adverse drug reactions: Definitions, diagnosis, and management. Lancet.

[5] Aabideen Z.U., Mumtaz M.W., Akhtar M.T., Mukhtar H., Raza S.A., Touqeer T., Saari N. (2020). Anti-obesity attributes; UHPLC-QTOF-MS/MS-based metabolite profiling and molecular docking insights of *Taraxacum officinale*. Molecules.

[6] Wen J.-J., Gao H., Hu J.-L., Nie Q.-X., Chen H.-H., Xiong T., Nie S.-P., Xie M.-Y. (2019). Polysaccharides from fermented Momordica charantia ameliorate obesity in high-fat induced obese rats. Food Funct..

[7] Yuan D., Huang Q., Li C., Fu X. (2022). A polysaccharide from Sargassum pallidum reduces obesity in high-fat diet-induced obese mice by modulating glycolipid metabolism. Food Funct..

[8] Zhang J., Kang M.-J., Kim M.-J., Kim M.-E., Song J.-H., Lee Y.-M., Kim J.-I. (2008). Pancreatic lipase inhibitory activity of *Taraxacum officinale* in vitro and in vivo. Nutr. Res. Pract..

[9] Raghu Mohan Rao P R.M.R.P., Jyothi Y., Rabban S. (2015). Anti-obesity activity of *Taraxacum officinale* in high fat diet induced obese rats. J. Chem. Pharm. Res..

[10] Pena-Leon V., Perez-Lois R., Seoane L.M. (2020). mTOR pathway is involved in energy homeostasis regulation as a part of the gut–brain axis. Int. J. Mol. Sci..

[11] Fernández-Veledo S., Vázquez-Carballo A., Vila-Bedmar R., Ceperuelo-Mallafré V., Vendrell J. (2013). Role of energy-and nutrient-sensing kinases AMP-activated Protein Kinase (AMPK) and Mammalian Target of Rapamycin (mTOR) in Adipocyte Differentiation. IUBMB Life.

[12] Shearin A.L. (2016). Akt Controls Adipocyte Function And Systemic Metabolism. Ph.D. Thesis.

[13] Kwan J.M. (2013). Akt Regulation of Adipogenesis: Implications for Skp2 Involvement. Ph.D. Thesis.

[14] Chondronikola M., Volpi E., Børsheim E., Porter C., Annamalai P., Enerbäck S., Lidell M.E., Saraf M.K., Labbe S.M., Hurren N.M. (2014). Brown adipose tissue improves whole-body glucose homeostasis and insulin sensitivity in humans. Diabetes.

[15] Kim S.H., Plutzky J. (2016). Brown fat and browning for the treatment of obesity and related metabolic disorders. Diabetes Metab. J..

[16] Xiang A.S., Meikle P.J., Carey A.L., Kingwell B.A. (2018). Brown adipose tissue and lipid metabolism: New strategies for identification of activators and biomarkers with clinical potential. Pharmacol. Ther..

[17] Yu G., Yang Z., Peng T., Lv Y. (2021). Circular RNAs: Rising stars in lipid metabolism and lipid disorders. J. Cell. Physiol..

[18] Zhou S., Wang Z., Hao Y., An P., Luo J., Luo Y. (2023). Dandelion polysaccharides ameliorate high-fat-diet-induced atherosclerosis in mice through antioxidant and anti-inflammatory capabilities. Nutrients.

[19] Finlin B.S., Zhu B., Confides A.L., Westgate P.M., Harfmann B.D., Dupont-Versteegden E.E., Kern P.A. (2017). Mast cells promote seasonal white adipose beiging in humans. Diabetes.

[20] Blüher M. (2019). Obesity: Global epidemiology and pathogenesis. Nat. Rev. Endocrinol..

[21] Li S., Xu Y., Guo W., Chen F., Zhang C., Tan H.Y., Wang N., Feng Y. (2020). The impacts of herbal medicines and natural products on regulating the hepatic lipid metabolism. Front. Pharmacol..

[22] Pamudurti N.R., Bartok O., Jens M., Ashwal-Fluss R., Stottmeister C., Ruhe L., Hanan M., Wyler E., Perez-Hernandez D., Ramberger E. (2017). Translation of circRNAs. Mol. Cell.

[23] Villiger A., Sala F., Suter A., Butterweck V. (2015). In vitro inhibitory potential of Cynara scolymus, Silybum marianum, *Taraxacum officinale*, and Peumus boldus on key enzymes relevant to metabolic syndrome. Phytomedicine.

[24] Hussain Z., Waheed A., Qureshi R.A., Burdi D.K., Verspohl E.J., Khan N., Hasan M. (2004). The effect of medicinal plants of Islamabad and Murree region of Pakistan on insulin secretion from INS-1 cells. Phytother. Res. Int. J. Devoted Pharmacol. Toxicol. Eval. Nat. Prod. Deriv..

[25] Yang Y., Ji J., Di L., Li J., Hu L., Qiao H., Wang L., Feng Y. (2020). Resource, chemical structure and activity of natural polysaccharides against alcoholic liver damages. Carbohydr. Polym..

[26] Chen Y., Fei S., Yu X., Tan M. (2023). Dandelion (*Taraxacum mongolicum*) Extract Alleviated H_2_O_2_-Induced Oxidative Damage: The Underlying Mechanism Revealed by Metabolomics and Lipidomics. Foods.

[27] Harb E., Kheder O., Poopalasingam G., Rashid R., Srinivasan A., Izzi-Engbeaya C. (2023). Brown adipose tissue and regulation of human body weight. Diabetes/Metab. Res. Rev..

[28] Aquilano K., Zhou B., Brestoff J.R., Lettieri-Barbato D. (2023). Multifaceted mitochondrial quality control in brown adipose tissue. Trends Cell Biol..

[29] Magro B.S., Dias D.P.M. (2024). Brown and beige adipose tissue: New therapeutic targets for metabolic disorders. Health Sci. Rev..

[30] Liu X., Zhang Z., Song Y., Xie H., Dong M. (2023). An update on brown adipose tissue and obesity intervention: Function, regulation and therapeutic implications. Front. Endocrinol..

[31] Bertoncini-Silva C., Zingg J.M., Fassini P.G., Suen V.M.M. (2023). Bioactive dietary components—Anti-obesity effects related to energy metabolism and inflammation. BioFactors.

[32] Ye Y., Liu H., Zhang F., Hu F. (2019). mTOR signaling in Brown and Beige adipocytes: Implications for thermogenesis and obesity. Nutr. Metab..

