# Supplementary figures and images for: Taraxacum Mongolicum Polysaccharides Reverses Mice Obesity via Activation of AKT/mTOR Pathway
Source: Nutrients. 2024 Sep 30;16(19):3330. doi: 10.3390/nu16193330 (PMC11478787; doi:10.3390/nu16193330)

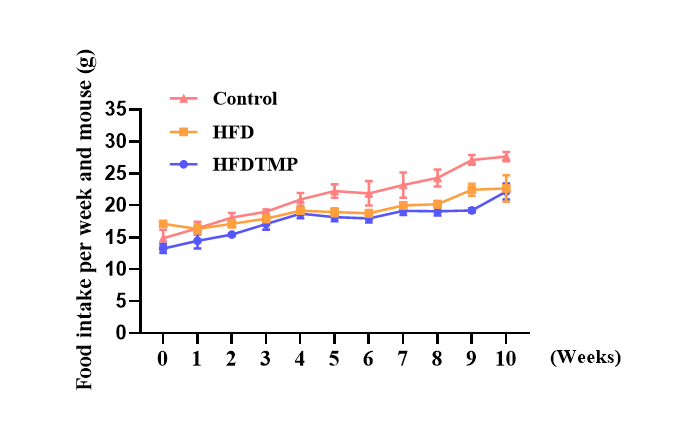

Supplement: Supplementary file 1 [file nutrients-16-03330-s001.zip › nutrients-3173423-supplementary.png]
